# Symptoms in Response to Controlled Diesel Exhaust More Closely Reflect Exposure Perception Than True Exposure

**DOI:** 10.1371/journal.pone.0083573

**Published:** 2013-12-16

**Authors:** Chris Carlsten, Assaf P. Oron, Heidi Curtiss, Sara Jarvis, William Daniell, Joel D. Kaufman

**Affiliations:** 1 Department of Environmental and Occupational Health Sciences, University of Washington, Seattle, Washington, United States of America; 2 Children's Core For Biomedical Statistics, Seattle Children's Research Institute, Seattle, Washington, United States of America; 3 Department of Environmental and Occupational Health Sciences, University of Washington, Seattle, Washington, United States of America; 4 Department of Medicine, University of Washington, Seattle, Washington, United States of America; University of California San Francisco, United States of America

## Abstract

**Background:**

Diesel exhaust (DE) exposures are very common, yet exposure-related symptoms haven’t been rigorously examined.

**Objective:**

Describe symptomatic responses to freshly generated and diluted DE and filtered air (FA) in a controlled human exposure setting; assess whether such responses are altered by perception of exposure.

**Methods:**

43 subjects participated within three double-blind crossover experiments to order-randomized DE exposure levels (FA and DE calibrated at 100 and/or 200 micrograms/m^3^ particulate matter of diameter less than 2.5 microns), and completed questionnaires regarding symptoms and dose perception.

**Results:**

For a given symptom cluster, the majority of those exposed to moderate concentrations of diesel exhaust do not report such symptoms. The most commonly reported symptom cluster was of the nose (29%). Blinding to exposure is generally effective. Perceived exposure, rather than true exposure, is the dominant modifier of symptom reporting.

**Conclusion:**

Controlled human exposure to moderate-dose diesel exhaust is associated with a range of mild symptoms, though the majority of individuals will not experience any given symptom. Blinding to DE exposure is generally effective. Perceived DE exposure, rather than true DE exposure, is the dominant modifier of symptom reporting.

## Introduction

We aimed to gain insight into the subjective experience of those exposed to DE, as exemplified by participants in controlled human exposure studies involving DE. Such studies have paralleled the momentum of DE as a model inhalant in the air pollution research community. Research that integrates subject’s perception of exposure in this setting has not been previously reported. Exploring subject perception is important in interpretation of controlled exposure studies, in which perception-related pathways (eg. those related to emotional stress) may theoretically confound or explain effects. Subject perception is also relevant to general public health and well-being, given the frequency of complaints attributable to DE or diesel fumes in some populations [1,2] and the increasing global extent of DE exposures. 

This study examined healthy subjects’ perceptions and reported symptoms in response to controlled diesel exhaust (DE) exposure and the association of these responses with baseline and pre-exposure subject characteristics (chemical intolerance and anxiety). The overall hypothesis was that symptoms resulting from exposure to inhaled DE, as compared to filtered air (FA), are more related to perceived exposure than to true exposure. 

## Materials and Methods

This project was approved by the University of Washington institutional research review board. Informed consent was obtained and the investigation was conducted according to the principles expressed in the Declaration of Helsinki.

Data reported were obtained in the context of experiments studying mechanisms underlying cardiovascular effects of air pollutants in humans, using inhaled diesel exhaust as a model exposure. The controlled environmental facility has been previously described [3]. In brief, exposure was generated via a 2002 turbocharged direct-injection 5.9 liter Cummins B-series engine (6BT5.9G6, Cummins, Inc., Columbus, IN) in a 100 kW generator set (Sommers, Ltd., Tavistock, Ontario) using ultra-low sulfur commercial diesel fuel with a load maintained at 75% of rated capacity by a load-adjusting bank (model LBS-B-100, Simplex, Springfield, IL). This exhaust was diluted approximately 400:1 in two stages and aged for approximately 5 minutes before entering the 110 m^3^ exposure room, with concentrations regulated via feedback control. Relative humidity and temperature were kept constant at 50% and 70 degrees Fahrenheit. The ventilation system was run for 1 hour with FA prior to beginning each exposure. Non-smoking adults aged 18-49, some of whom were recruited based on having metabolic syndrome[4], were recruited via advertisements at the University of Washington and the surrounding community. After an initial phone screen, eligible subjects were invited to come to the lab for an in-person screening session that included: an explanation of the experimental protocol, a facility tour, informed consent, questionnaire, anthropometric assessment, and spirometry. Except within the study focused on the metabolic syndrome, subjects were excluded if chronic health conditions, including hypertension, asthma, diabetes, or hypercholesterolemia, were identified. 

Forty-six individuals participated in three experiments over 2004 to 2010, using the same overall exposure and questionnaire protocols. The first two experiments (hereafter, “Experiments 1 and 2”) consisted of three visits – one each with filtered air (FA; hereafter “none”), DE titrated to 100 µg/m^3^ particulate matter of diameter less than 2.5 microns (PM_2.5_) hereafter referred to as “medium”, or DE titrated to 200 µg/m^3^ PM_2.5_ (hereafter “high”). The third experiment (hereafter, “Experiment 3”) included four visits, two each with FA and high DE exposure, and with patients taking either antioxidants or placebo capsules, in such a manner that each visit reflected one distinct combination of the two options (FA/high; antioxidant/placebo). Concentrations of particulate matter and concomitant gases, were monitored in real time using a system previously described [3]. Levels of nitrogen dioxide (NO_2_) were approximately 0.035 parts per million. Exposure to each of the levels was for 2 hours and occurred for each subject in a fashion randomized and counterbalanced with regard to order. Subjects were seated (did not exercise) during the exposures. All personnel in contact with subjects were blinded to the exposure level in place for that testing session. A washout period (at least 2 but no greater than 4 weeks) occurred between exposures for participants. Women’s exposures were timed to the follicular phase of their menstrual cycle. Subjects fasted overnight or for at least ten hours. Subjects confirmed that they were experiencing no symptoms of illness on the morning of the session. Of the 46 individuals participating in Experiments 1-3, there were 43 participants with and at least one DE and one FA session and both pre- and post-exposure questionnaires within each session, and this analysis includes only those 43 individuals (total 143 sessions [83 DE and 60 FA]).

For each experiment, the protocol mandated that participants answer a self-paced Palm Pilot questionnaire about symptoms at each of 6 stages: baseline (before exposure initiation; “pre”), within the first hour of exposure (“early”), at the end of exposure (“late”); 6 hours after the baseline (“afternoon”); 12 hours after baseline (“evening”); and the following morning approximately 24 hours after exposure initiation (“next”). Subjects were asked about the presence of symptoms within 4 symptom *clusters* ("chest" = chest shortness of breath?, chest tightness? chest pain? chest wheezing/whistling sounds? throat dry scratchy or sore?; "nose" = nose itchy?, nose blocked?, nose running? nose painful or stinging? sneezing?; "eye" = eyes itchy? eyes watering? eyes painful or stinging?) as well as 3 individual symptoms ("headache"; "fatigue"; "nausea"). The symptoms queried were chosen as those commonly reported in the literature [5–8]. To reduce multiple comparisons and to allow a ‘snapshot’ of clinical pertinence, analysis was performed at the level of the symptom cluster, rather than individual symptom for the 4 clusters above, while headache, fatigue, and nausea were each evaluated independently. Regarding perception, subjects were asked at the end of each exposure, “What exposure level do you think you received during this exposure session?” (None, Medium, High, or Don’t Know).

### Data analysis

All analyses were performed in R version 2.13.0. P-values <0.05 were considered significant and p-values of 0.05-0.10 were considered of borderline significance. Because there were very few reports of symptom severity greater than mild (see Results), symptomatology was thereafter simplified to a dichotomy (yes/no) for each cluster. Correct perception was defined as either perception of exposure to diesel (either DE_100_ or DE_200_) in the context of either level or perception of FA in the context of FA, with “don’t know” being scored as incorrect. After descriptive analysis, we carried out a bootstrap-null significance test for blinding (described below), and Poisson and logistic mixed-effects models for the effects of exposure and perception, with participant and the contrast between high-symptom stages and low-symptom stages as the grouping variables. 

## Results

As noted in Table 1, subjects were mostly male Caucasians whose mean BMI reflects the presence of metabolic syndrome in a subset of the subjects.

**Table 1:** Demographic data on 43 subjects.

Age†		32.9±9.7 *
BMI		29.8±10.0 *
Gender	Female	16 (37%)
	Male	27 (63%)
Ethnicity	Caucasian	35 (81%)
	Hispanic	3 (7%)
	African American	3 (7%)
	Other	2 (5%)
Metabolic Syndrome	Yes	16 (37%)
	No	27 (63%)

^*^ mean ± standard deviation

^†^ age at enrollment

**Figure 1:**
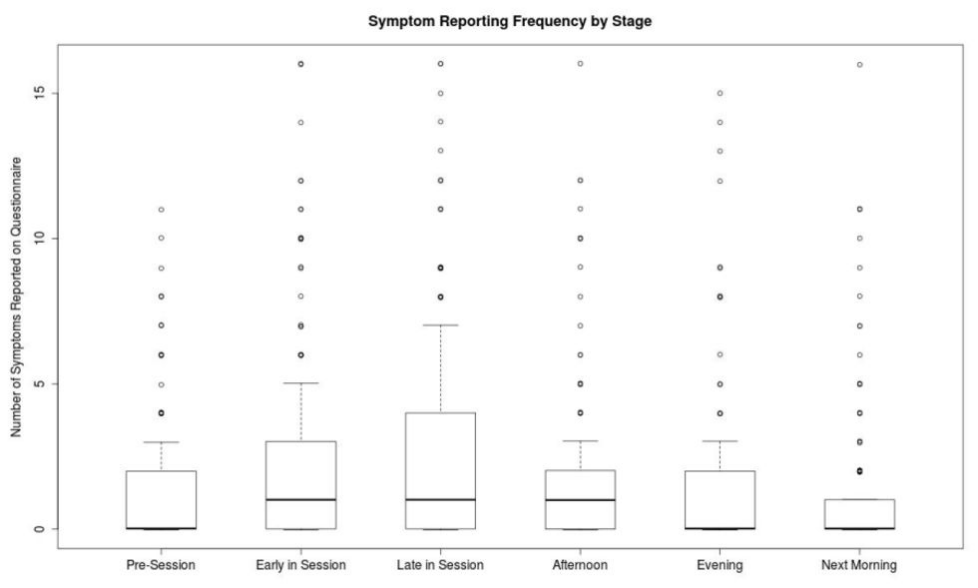
Symptom reporting frequency by stage.

As noted in Table 2, symptoms were typically absent; when present they were typically mild. Fatigue was the most commonly reported symptom; nausea was the least commonly reported. Since severities worse than mild were very rare, subsequent analysis dichotomized clusters such that positive response to at least one question within the cluster led to that cluster being considered positive. Symptom reporting peak had plateaued by approximately 6 hours following DE exposure (Figure 1) and so that time point became the focus in subsequent analyses (coincidentally, “afternoon” was thought *a priori* as a reasonable balance between early and late timepoints, as the primary timepoint for comparison to baseline). 

**Table 2:** Prevalence of reported symptom (headache, nausea, fatigue) or cluster severity across all visits and stages, by symptom/cluster.

**Type**	**None**	**Mild**	**Mild-Moderate **	**Moderate **	**Moderate-Severe **	**Severe**
**Headache**	79.1%	15.7%	3.0%	1.4%	0.7%	0.1%
**Nausea**	93.2%	5.0%	1.4%	0.5%	0.0%	0.0%
**Fatigue**	68.1%	20.8%	7.1%	3.3%	0.6%	0.1%
**Eyes**	78.6%	14.3%	5.3%	1.5%	0.2%	0.0%
**Nose**	70.6%	22.2%	3.7%	2.2%	0.9%	0.4%
**Throat**	79.9%	14.3%	4.4%	0.8%	0.5%	0.1%
**Chest**	86.8%	9.8%	2.1%	0.8%	0.2%	0.2%

For each individual at a given stage contributing to this table, the severity is based on the highest severity amongst all questions within the given cluster.

Though 58 percent of all exposures were truly DE, 56 percent of exposures were perceived to be DE; of all 82 exposures to DE, 53 (65 percent) were correctly perceived as DE (Table 3; one subject excluded from this particular analysis, as noted). For subsequent analyses of blinding, the “Medium” and “High” guesses were grouped together to create a dichotomous “DE” vs. “FA” variable. The overall rate of correct perception was 61.2%. Nine of 43 participants correctly identified all their sessions as DE or FA. Simply guessing DE for all exposures would have yielded a 66.7% success rate in Experiments 1 and 2 (where 2 of 3 exposures were to DE), so the observed rate of successful perception is not an indication that participants were unblinded. 

**Table 3:** Participant perceptions of exposure versus true exposure, as assessed by the questionnaire administered during the exposure.

Perceived exposure					% correct
True Exposure	"HIGH"	"MEDIUM"	"NONE"	**Total**	
FA	7	19	34	**60**	**57**
100	3	12	6	**21**	65*
200	11	27	23	**61**	
**Total**	**21**	**58**	**63**	**142**	**61**

N = 42 (one subject’s perception record was missing). * 100 and 200 combined.

**Figure 2:**
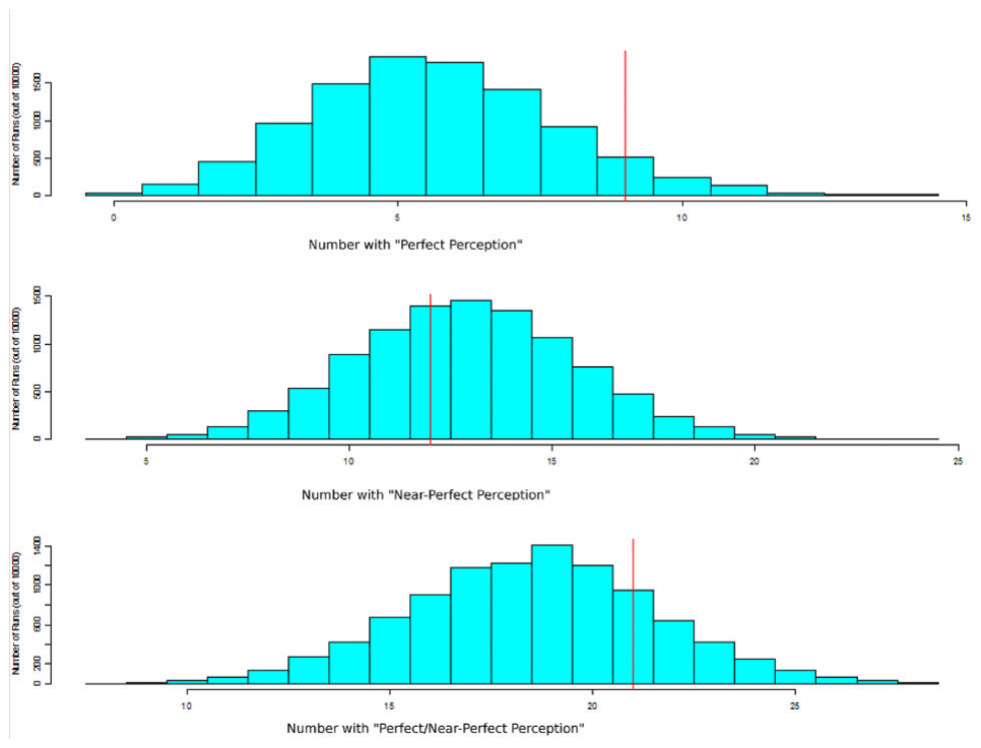
Bootstrap analysis to test the null hypothesis that any association between perception and exposure is due to chance. The blue bars represent the numerically-generated bootstrap null distribution for each scenario (A: perfect perception (correct for each exposure for a given individual); B: near-perfect perception (incorrect on only one exposure); C: those with either perfect or near-perfect perception). The red line represents the true frequency of each scenario from our questionnaire-based observation.

To better ascertain the degree of effective blinding, we created a numerically-generated bootstrap null distribution. The null hypothesis is that any association between perception and exposure is due to chance, recognizing that each person is allowed a unique perception pattern. This null distribution is equivalent to bootstrap resampling, stratified by participant. As Figure 2a shows, the number with “perfect perception” (vertical red line in top frame) is greater than the middle of the distribution, but not significantly so (p=0.09). “Near-perfect perception” participants (12 subjects who had only one perception misaligned with true exposure; Figure 2b) fits well within the null hypothesis of effective blinding. Figure 2c combines those with “perfect perception” with those with “near-perfect perception” (total of 21 subjects). This data suggests that a trend to unblinding was not statistically significant

As noted in Table 4, the effect of perceived appears stronger than the effect of true exposure in symptom reporting; perception, but not exposure, has a significant effect at the cluster level. 

**Table 4:** Effect of diesel exhaust exposure, or perception of diesel exhaust exposure, on symptom or symptom cluster.

	**Diesel Effect**			**Perception Effect**		
**Symptom (Cluster)**	**Positive**	**OR**	**Woolf**	**Positive**	**OR**	**Woolf**
Any Chest Symptom	27	1.7	0.58	12	27.5	0.06
Any Nose Symptom	50	1.3	0.64	28	4.7	0.03
Any Eye Symptom	43	1.5	0.48	25	3.6	0.13
Headache, Fatigue or Nausea	70	1.4	0.55	41	3.4	0.06

Positive = number of sessions, amongst column total, in which symptoms were reported as present

OR = differential odds-ratio: the ratio between the odds-ratio post-exposure and the odds-ratio pre- exposure (each of these odds-ratio measuring odds of given symptom cluster for DE exposure relative to FA exposure, or [for “perception effect”] for *perceived* DE exposure relative to perceived FA exposure). Note: for calculating the odds-ratio, 0.5 was added to each cell count, in order to avoid division by zero.

Woolf = the asymptotic Chi-Squared p-value reported by the Woolf test.

## Discussion

Symptom data from available diesel exhaust exposure studies is sparse, with much of the data coming from case reports lacking detailed information of exposure concentrations [5]. Our study is important in systematically assessing symptomatic responses to a controlled DE exposure at varying exposure concentrations; in a position statement, the American Thoracic Society considered such symptoms to be significant adverse effects of air pollution if they are severe enough to interfere with normal activities [9]; while we did not specifically assess interference with normal activities, we have added significantly to the literature by carefully quantifying the severity of these symptoms in the setting of acute exposure to DE, and the symptoms are generally mild. 

While a prior study made a limited assessment of symptomatic response to controlled DE [7], ours is the first to formally report the effectiveness of blinding and the first to relate perceived exposure to symptomatology. Our analysis points to three main findings that contribute to the literature regarding exposure to diesel exhaust, particularly in the controlled exposure setting: 1) for a given symptom cluster, the majority of those exposed to moderate concentrations of diesel exhaust do not report such symptoms; when they do, the vast majority of complaints are mild in severity; 2) blinding to exposure is generally effective; 3) perceived exposure, rather than true exposure, is the dominant modifier of symptom-reporting. 

Rudell and colleagues [7] note that the presence of a particulate trap did little to change nasal symptoms; however, NO_2_ levels in the report from Rudell and colleagues are approximately 2 orders of magnitude higher than those noted in our study. In our study, NO_2_ levels are uniformly at least one full order of magnitude below the apparent NO_2_ odor threshold (0.5 ppm; [10]), making it seem unlikely, at first glance, that symptoms are attributable to NO_2_. However, as the nose is the sentinel of soluble irritants, it is possible that even relatively low levels of NO_2_ are perceivable, especially to those with chemical odor intolerance and/or “sensory hyperreactivity” [11]. 

Given that the more prevalent symptoms were associated with mucous membranes, it is reasonable to also consider aldehydes in DE, since aldehydes are known mucosal irritants; some aldehyde species are typically measurable at all exposure levels. It is notable that formaldehyde levels (approximately 10-40 μg/m^3^; unpublished data) obtained in our laboratory are considerably lower than those of the most similar study to date [7]. Such levels are consistent with levels found in trucking settings [12], but considerably below the odor threshold suggested in the literature (approximately 120 μg/m^3^; [13]). Further, the irritation threshold for formaldehyde appears higher than the odor threshold [13,14]. However, given inter-individual variability in upper airway sensory irritation [15], it is conceivable that those more chemically intolerant in general are symptomatic at aldehyde levels approximately one order of magnitude below the odor threshold for the general healthy population [16,17]. There is evidence for respiratory symptomatology in children at formaldehyde levels approximately one order of magnitude lower than those in our study [18]. 

Our finding that subjects seem sufficiently blinded to exposure level is important. Although some outcome measures of interest in controlled exposure studies might be considered unaffected by knowledge of exposure, blinding remains an important element of experimental methods and our results are reassuring that unblinding is a very unlikely explanation for findings associated with DE in our model. The effective blinding may be due to the observation that a residual odor, characteristic of diesel exhaust but otherwise at ambient levels of PM, is present typically in the exposure area even on FA exposure days.

The most important potential limitation to this study may be small sample size. However, our sample size is large for this type of study (controlled human crossover of exposure to air pollution), and it should be noted that the crossover nature of this study inherently decreases the sample size necessary to detect a given effect, even relative to other randomized study designs [19]. 

Controlled human exposures to inhaled pollutants play an important role within the breadth of investigation into health effects of air quality. They have been particularly influential in providing biological plausibility to finding from epidemiological and animal-based research. In that context, it is important to ensure that such human exposures are effectively blinded, since the mere intention to blind is not sufficient. We believe that our study is the best effort to date to carefully assess the effectiveness of blinding, and it makes an important contribution to the literature by showing that blinding is effective since symptoms are not driven by the true exposure conditions.

## Conclusion

Controlled human exposure to moderate-dose diesel exhaust is associated with a range of mild symptoms, though for the majority of individuals will not experience any given symptom. Blinding to DE exposure is generally effective. Perceived DE exposure, rather than true DE exposure, is the dominant modifier of symptom-reporting. Overall, this lends reassurance to the validity of symptom-independent and sub-clinical findings associated with DE exposure in controlled human models.
